# Linking rare variants to cell-type function in profound autism with brain transcriptomics and foundation models

**DOI:** 10.1016/j.xgen.2026.101192

**Published:** 2026-04-08

**Authors:** Alma Dubuc, Thomas Renne, Guillaume Huguet, Sébastien Jacquemont, Tomasz Nowakowski

**Affiliations:** 1 Department of Neurological Surgery, University of California San Francisco, San Francisco, CA 94143, USA; 2 École Normale Supérieure de Lyon, Université Claude Bernard Lyon 1, Université de Lyon, 69342 Lyon Cedex 07, France; 3 Program in Computational Biology and Bioinformatics, Yale University, New Haven, CT, USA; 4 Centre Hospitalier Universitaire Sainte-Justine Research Center, Montreal, QC, Canada; 5 Department of Biochemistry and Molecular Medicine, Université de Montréal, Montréal, QC, Canada; 6 Department of Pediatrics, University of Montreal, Montreal, QC, Canada; 7 Weill Institute for Neurosciences, University of California, San Francisco, San Francisco, CA 94158, USA

## Abstract

Genetic association studies have identified numerous genes harboring protein-disrupting variants in individuals with profound autism, but identifying convergent points of vulnerability remains challenging. We discuss how brain transcriptomic resources help decode the cellular consequences of these rare gene-disrupting variants. The functional interpretation of genetic associations has largely relied on gene ontologies and protein-interaction networks, with newer approaches leveraging single-cell expression to estimate cellular enrichment. However, the broad expression of many autism-associated genes confounds cell-type-specific effects. We therefore propose a framework quantifying the trade-off between a gene’s cell-type specificity and sensitivity. The limited overlap between genetic associations and transcriptomic alterations in autistic brains prompts a discussion about causality. We examine whether foundation models linking genetic variation to cell-type transcriptomes could clarify the cellular functions affected by autism-associated variants. By combining experimental perturbations, artificial-intelligence-driven inference, and *postmortem* validation, we propose a unifying mechanistic framework for rare-variant liability in autism.

## Graphical abstract

**Figure:**
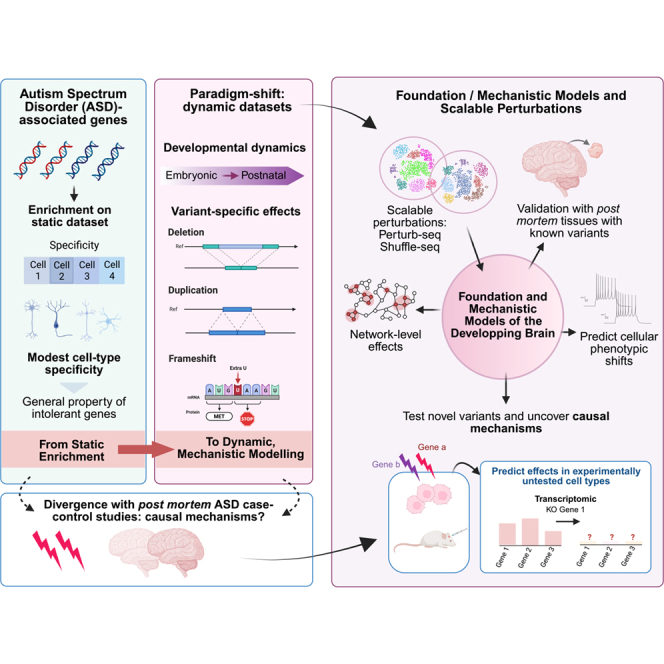

## Teaser

Dubuc et al. synthesize genetic and single-cell evidence to reassess biological convergence in autism. They propose that foundation models of the developing brain, integrating scalable perturbation data with *postmortem* validation, could bridge genetic variation to brain circuits and provide a predictive framework for neurodevelopmental disorders.

## Introduction

The human brain consists of approximately 100 billion neurons distributed across hundreds of anatomical and functional regions.^1^^,^^2^ For decades, cell-type distinctions were based on anatomical position, morphology and projection patterns, or neurotransmitter character, but recent advances in single-cell and single-nucleus RNA sequencing (scRNA-seq and snRNA-seq) have defined thousands of cell types based on patterns of gene expression in the adult and developing mammalian brain.^3^^,^^4^^,^^5^^,^^6^^,^^7^^,^^8^^,^^9^

The genetic risk architecture of autism spectrum disorder (ASD) is complex and often overlaps with that of related neurodevelopmental disorders (NDDs), including intellectual disability, speech and language disorders, attention-deficit hyperactivity disorder (ADHD), and epilepsy, which frequently co-occur with autism.^10^ Rare gene-disrupting variants contributing to ASD^11^^,^^12^ have implicated dozens of genes in autism vulnerability but provided remarkably limited insights into convergent biological mechanisms, due to pleiotropic biochemical functions and strong genetic constraint.^12^^,^^13^^,^^14^ Transcriptomic datasets have been used to identify brain regions, developmental periods, and cell types with the strongest co-expression of ASD-associated genes. However, the broad expression profiles of most autism-associated genes present a challenge in predicting the consequences of any specific variant in each of the cell types that express the gene.^11^^,^^12^^,^^15^^,^^16^^,^^17^ To address this challenge, we propose a framework to quantify the trade-off between the specificity and the sensitivity of a gene for discriminating cell types.

Moreover, emerging experimental techniques, such as Perturb-seq and Shuffle-seq, provide scalable approaches to systematically measure the effect of gene haploinsufficiency on cellular transcriptomes.^18^^,^^19^^,^^20^^,^^21^ Despite these advances, datasets providing direct cellular-level observation of transcriptomic changes resulting from autism-associated gene-disrupting variants are still emerging. Large language models provide an exciting opportunity to complement these early datasets, helping to infer patterns and prioritize hypotheses. In particular, single-cell foundation models are pre-trained on massive single-cell datasets and can be deployed for downstream tasks such as perturbation predictions without retraining. They can further leverage Perturb-seq datasets to predict the impact of genetic variants on cellular transcriptomes, and, in the near future, on electrophysiological properties and morphology.^22^ Such methods provide a new avenue for understanding the mechanistic impact of rare autism-associated variants.

## Transcriptomic architecture of cell types

**Figure 1:**
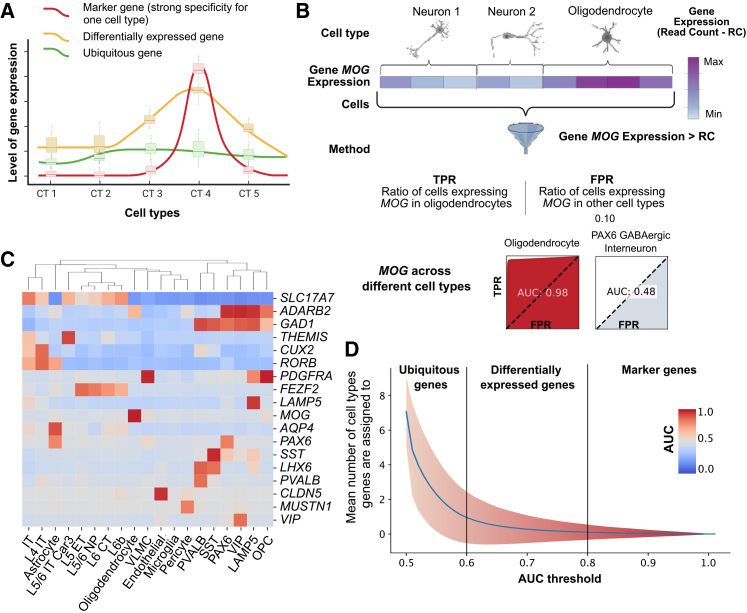
AUC-based framework to quantify cell-type specificity (A) Schematic representation of three types of gene expression across cell types: in red, a marker gene with a specific peak of expression in cell type 4 only; in orange, a DE gene with a basal expression across all cell types and an enrichment in cell type 4; and in green, a ubiquitous gene with a uniform gene expression across all cell types. (B) Illustration of the AUC calculation for the gene *MOG* in oligodendrocytes (and an illustration of the result for PAX6 GABAergic interneurons). For varying read count (RC) thresholds, the true-positive rate (TPR) is defined as the proportion of oligodendrocytes expressing *MOG* above a given threshold, and the false-positive rate (FPR) is defined as the proportion of non-oligodendrocyte cells expressing *MOG* above the threshold. The receiver operating characteristic (ROC) curve is obtained by plotting FPR on the *x* axis and the TPR on the *y* axis. The AUC (red surface) quantifies the trade-off between sensitivity (TPR) and specificity (FPR) for the gene in the cell type of interest. For the *MOG*-oligodendrocyte pair, a high AUC close to 1 indicates high cell-type specificity, whereas, in PAX6 GABAergic interneurons, a much lower AUC (gray surface) approaching 0.5 corresponds to a low specificity. (C) Heatmap representing the AUC of 18 canonical marker genes (in rows) for 19 subclasses^25^ (in columns). The AUC is color coded from blue to red: a pair in blue indicates that the gene has a low specificity for the cell type; a pair in red indicates a specific expression in the cell type. Cell types are clustered based on the markers’ AUCs. (D) Plot of the mean number of cell types genes are assigned to, depending on their AUC (blue line). The area represents the standard deviation and is color coded following the AUC value defined in (C).

Single-cell gene-expression and epigenomic datasets have begun to reveal complex taxonomies of cell types in the mammalian brain.^2^^,^^3^^,^^4^^,^^7^^,^^8^^,^^9^^,^^23^ These cells can be hierarchically organized into classes (e.g., excitatory glutamatergic, inhibitory GABAergic, and glial), subclasses (e.g., vasoactive intestinal polypeptide [VIP]-expressing, somatostatin [SST]-expressing, lysosomal associated membrane protein family member 5 [LAMP5]-expressing interneurons), types (e.g., SST-CALB2, SST-CHODL, LAMP5-LHX6), and subtypes.^3^^,^^24^ Gene-expression profiles can be ranked on a continuum from very high to low specificity for any given cell population. Marker genes are highly specific, while housekeeping/ubiquitous genes are expressed across all cell types. However, most coding genes are preferentially enriched in a few specific groups of cells. The latter are also known as differentially/preferentially expressed or enriched genes. (Figure 1A).

Marker genes show quasi-categorical expression with transcripts specifically detected in a single cell class, type, or subtype. For example, *GAD1* is a marker gene for GABAergic classes, while *VIP* and *SST* are marker genes for respectively VIP and SST subclasses.^25^ Within the SST subclass, the marker gene *CHODL* defines the group of long-range projecting (LRP) interneurons (SST-CHODL or SST-LRP).^26^ Such genes are specific and represent the simplest case for predicting cellular vulnerabilities to genetic variants. When a gene is exclusively expressed in a cell type, it can be assumed that it is implicated in some aspect of the biology of that cell type.

By contrast to marker genes, differentially expressed (DE) genes are preferentially expressed by multiple cell populations. This enables an alternative way of defining cellular identities: not by unique marker expression, but through overlapping combinations of genes whose individual expression may be shared across distinct classes or subclasses (Figure 1A).

Single-cell/nucleus RNA sequencing (RNA-seq) studies have focused on methods to identify significant DE genes, but they do not quantify the cell-type specificity/sensitivity of genes. As a result, distinguishing the boundaries between marker, DE, and ubiquitous genes remains challenging. The accuracy of gene expression to discriminate between cell types can be fully captured by the receiver operating characteristic (ROC) curve, which takes into account two metrics: sensitivity (the fraction of cells within a cell type in which transcripts of a gene of interest are detected) and specificity (the fraction of cells outside the cell type in which the transcripts of interest are detected). The resulting ROC represents a trade-off between sensitivity and specificity computed for multiple thresholds (different read counts for transcripts of a given gene). The area under the ROC curve (AUC) is a numerical value that quantifies the trade-off between specificity and sensitivity across different expression thresholds (Figure 1B). A gene with a high AUC value (close to 1) for a given cell type could meet criteria for a marker gene: it would be expressed in most cells of the cell type (high sensitivity) and in no other cells (high specificity). In contrast, a gene with an AUC of 0.5 would be nonspecific and could be considered ubiquitous.

To validate this approach, we computed the AUCs of all protein-coding genes across 19 cortical cell subclasses using transcriptomic data from the Allen multi-cortical snRNA-seq data.^25^ All 18 canonical marker genes^25^ used at the subclass level have values above 0.8 for their corresponding subclasses. We therefore used 0.8 as a definition for “candidate marker” genes (Figures 1B and 1C). We observed that the three cell classes were well predicted by an average of 42 candidate marker genes per class (total of 127). When 110 cell clusters are considered, we identified on average 20 candidate marker genes per cluster (for a total of 2,253 marker genes, ∼10% of the human coding genome). Because the number of candidate marker genes assigned to cell types at the highest levels of granularity decreases, expression profiles across many genes (less specific than marker genes) may be required to discriminate highly granular cell types.

It is reasonable to assume that between highly specific candidate marker genes (AUC ≥ 0.8) and ubiquitous genes (AUC < 0.6) lies a large proportion of genes that show preferential expression across a few cell types, representing a substantial contribution to the transcriptomic architecture of cell types. The AUC provides a continuous value along a gradient ranging from marker to DE to ubiquitous (Figure 1D). Strategies similar to the AUC framework, such as the specificity index, ^27^ can also be used to assess gene specificity.

Gene-expression variation within a cell class/subclass/type is influenced by factors such as cell state, spatial localization, and extrinsic conditions (e.g., stress, oxygen availability, immune environment). Weighted Gene Co-expression Network Analysis (WGCNA) is an analytical framework that models these axes of variation of expression by grouping genes with correlated patterns of expression into “modules.”^28^ Early applications of WGCNA to bulk RNA-seq measurements leveraged correlated variation of cell-type proportions to extract modules of cell-type-specific gene expression.^29^ Recent implementations of WGCNA to single-cell data can additionally capture regulatory programs and cell-state transitions irrespective of tissue-level compositional effects.^30^

## Cellular programs altered by autism-associated gene-disrupting variants

ASD and related NDDs have a high estimated heritability (0.65–0.91 for ASD),^11^^,^^12^^,^^31^^,^^32^^,^^33^ and understanding mechanisms underlying genetic liability could provide a window into causal mechanisms.^11^^,^^12^^,^^15^^,^^17^^,^^34^ The majority of recent studies have focused on understanding how rare variants disrupt genes and their downstream effects on biological processes (at the cellular and tissue levels). Functional annotation of ASD-associated genes remains very incomplete.^35^ In particular, many of the genes associated with autism and related NDDs have pleiotropic effects, making it difficult to prioritize and distinguish ASD-associated biological functions.

Functional liabilities can be interpreted at different levels of brain organization. At the subcellular level, molecular functions have been organized into signaling pathways and cellular processes that are defined by ontologies, such as the manually curated Gene Ontology (GO),^36^^,^^37^ and within experimentally driven gene ontologies such as WGCNA modules.^28^ At the tissue level, we can examine how specific cell types are relevant to circuits^38^ and behaviors.^39^ Emerging single-cell-resolution datasets provide a new opportunity to extend the analysis of ASD-associated gene convergence.

### Rare-coding-variant architecture of autism and associated NDDs

While the genetic architecture of autism includes common and rare variants (frequency below 1/1,000),^11^^,^^12^^,^^33^^,^^40^^,^^41^^,^^42^^,^^43^ the interpretability of genetic studies has been more fruitful in the case of rare gene-disrupting variants. These high-effect-size variants are routinely identified in the pediatric clinics and are prime candidates for developing gene therapies and other therapeutic interventions^44^^,^^45^^,^^46^ targeting specific molecular mechanisms. Individual variants in ASD-linked genes can have classes leading to a broad range of molecular effects. Nonsense and frameshift classically lead to loss of function (depending on whether nonsense-mediated decay occurs), but may also have other deleterious effects (e.g., due to a truncated protein). Missense and splice-site variants may also lead to loss or gain of function. Copy-number variants (CNVs) (i.e., duplications and deletions) are usually associated with increases or decreases in gene expression, respectively (when they fully encompass genes).

Previous studies associating rare variants with ASD have mostly relied on statistical methods based on the null distribution of *de novo* variants across the coding genome, because current sample sizes are still underpowered for rare-variant case-control association studies.^11^^,^^12^^,^^41^ Such studies have identified 405 high-confidence ASD genes (Simons Foundation Autism Research Initiative [SFARI] class 1 and syndromic release Q1 2024), including a category of genes with a broader syndromic/neurodevelopmental presentation, referred to as syndromic.^11^^,^^12^^,^^42^

**Figure 2:**
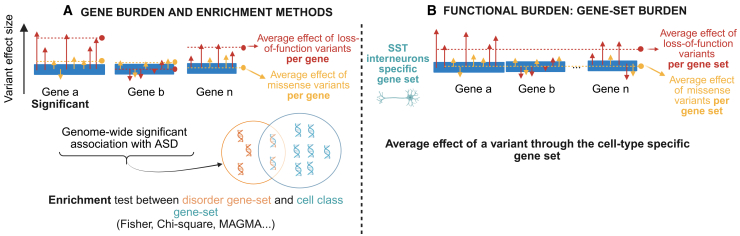
Comparison of two frameworks associating gene-disrupting variants to cell types (A) The gene-burden approach averages the effects of all variants impacting a given gene. The output is therefore an average effect size per gene. Genes that reach genome-wide significance are compiled into a list, which is then analyzed using single-cell databases to test for cell-type enrichment. This identifies cell types significantly enriched in genes associated with the disorder. (B) The functional-burden approach averages the effects of all variants impacting a gene set (e.g., the set of genes assigned to a given cell type). By pooling across gene sets, this method accounts for a larger number of variants, thereby increasing statistical power. The output is therefore an average effect size per gene set. This figure is inspired by Weiner et al.^48^

Most ASD-associated genes have been identified using gene-level burden analyses, aggregating all classes of disrupting variants within each gene^11^^,^^12^ (Figure 2A). This approach assumes that variants in a given gene are associated with homogeneous effects (magnitude and directionality). This assumption overlooks the fact that different classes of variants disrupting the same gene can differ substantially in their consequences: for instance, loss-of-function and missense mutations may have opposite effects on protein structure or function.^47^ Even variants of the same class may vary in their molecular effects. For example, stop mutations may lead to a truncated protein or nonsense-mediated decay (NMD) based on their position. While NMD leads to loss of function, truncated proteins may have gain-of-function effects. An important frontier will require functional validation of these variants, including those that may have opposite molecular effects on protein structure. In particular, whether different classes of variants map onto different phenotypic presentations of ASD remains uncertain.

Overall, ASD-associated genes are under high genetic constraints (variants disrupting such genes have low genetic fitness and are therefore rare in the general population).^12^^,^^13^^,^^14^ As a result, measures of genetic constraint have been used to prioritize candidate genes potentially associated with autism. Many studies have demonstrated that, beyond the well-replicated ASD-associated genes mentioned above, constrained genes are, as a group, associated with ASD.^16^^,^^49^ Loss-of-function observed/expected upper-bound fraction (LOEUF) score is a well-established measure of genetic constraint^50^ based on the tolerance of a gene to loss-of-function variants (under-constraint genes, LOEUF < 0.6), which we will refer to in several subsequent sections of this review. Overall, our knowledge of the genetic architecture of ASD is highly biased toward *de novo* variants, reflecting limited statistical power to detect associations involving inherited disruptive variants in more mildly constrained genes.

Genetic studies have sought to increase statistical power by aggregating *de novo* and inherited variants (burden methods). The most common burden approach at the gene level (implemented in pipelines such as SKAT^51^) is still underpowered to detect many associations. To increase power, burden approaches have been extended to gene sets defined by shared biological functions^17^^,^^52^(Figure 2B). This functional-burden method assumes that genes implicated in the same function will share similar effects (level and directionality of effects) on a phenotype. A functional burden therefore quantifies the effect of a gene set (instead of a single gene) on a phenotype, providing the mean effect size of variants disrupting this gene set. The increased power provided by functional burden allows different classes of variants, such as deletions, duplications, stop-gain, frameshift, missense, or splice-site variants, to be studied separately. A significant challenge of any burden analysis is that variants with heterogeneous effects (e.g., opposing/mixed effects) may cancel each other out when their effects are aggregated/averaged across individuals.^51^ This risk increases when aggregating variants across gene sets^48^ (Figure 2). Alternative methods for analyzing variance (e.g., SKAT-O) improve power when genetic variants have mixed effects or when a large proportion of them are non-causal.^53^

The functional burden was recently used to study the association between CNVs and single-nucleotide variants disrupting functional gene sets (assigned to brain cell types and cortical regions) and psychiatric disorders, including autism, related NDDs, schizophrenia, bipolar disorder, ADHD, major depression, as well as cognitive ability, which is frequently affected in these conditions.^17^^,^^52^^,^^54^ This approach enabled the direct association of ASD and cognitive ability with cell-type-related genes disrupted by CNVs. Because functional burden was conducted for deletions and duplications separately, the results highlight that most associations are specific to one or the other of these two opposing gene dosage alterations. As an example, cognition was impacted by duplications of genes expressed in excitatory deep-layer neurons and deletions of genes expressed in glial cells. These results show that the effects on a neurodevelopmental trait of genes and corresponding cell types are dependent on the class of variant. Similarly, a significant association of excitatory prenatal neurons with ASD was reported for deletions specifically.^52^ Furthermore, a recent functional-burden study of ASD demonstrated that loss-of-function variants preferentially mapped to prenatal cell types, while missenses and duplications were linked to postnatal excitatory neurons.^54^

Overall, functional-burden methods can reveal a robust association between phenotypes and cell-type-specific gene sets, even when individual variants or genes do not (yet) meet criteria for gene-wide significance.

### Convergence of ASD-associated genes on cell types and function

Understanding how genetic variants associated with ASD affect cell functions across development represents a central question. Most studies have used cell-type-specific expression-enrichment analysis to functionally decode genetic variants associated with ASD.^15^ They followed a multistep approach: (1) establish gene-level association (aggregating all classes of gene-disrupting variants) with ASD and (2) perform an enrichment analysis of genome-wide significant genes across cell types (Figure 2A). Studies assess cell-type enrichment by either testing whether associated genes show higher expression in a given cell type^12^ or testing over-representation of cell-type-specific gene sets.^55^

Such approaches demonstrated an enrichment of high-confidence ASD-associated genes in maturing excitatory,^11^^,^^12^^,^^34^^,^^56^^,^^57^^,^^58^ as well as inhibitory, neurons,^11^^,^^12^^,^^58^^,^^59^ as opposed to glial cells. However, enrichment analyses have not identified specific neuronal subtypes. In some analyses, enrichment among deep-layer glutamatergic neurons was detected,^11^^,^^57^ yet others showed enrichment among superficial intratelencephalic cortical neurons.^34^^,^^60^ Similarly, autism studies have not identified specific subtypes of GABAergic neurons: while some analyses have noted subtypes of VIP and SST interneurons as being enriched for genes disrupted by the 16p11.2 microdeletion,^61^ their findings have thus far not generalized to other ASD-associated variants.

These cell-type enrichment studies have not accounted for the diversity of autism phenotypes and comorbidities. While there is no evidence for ASD-specific genes,^62^ studies have attempted to distinguish genes preferentially associated with autism from those mainly associated with broader neurodevelopmental phenotypes. Only a small subset—36 and 14 genes in two studies^11^^,^^63^—have been classified as predominantly associated with ASD. Even in this subset, some genes confer risk for schizophrenia^11^ and ADHD^64^ (e.g., *DSCAM*, *ANK2*, and *NRXN1*). Although ASD-specific genes remain elusive, it is plausible that genes that contribute preferentially to ASD may map to distinct cell-type profiles. Such an analysis has shown that ASD-predominant genes were broadly enriched in maturing excitatory neurons, whereas NDD-predominant genes were enriched in less differentiated cell types (both excitatory and inhibitory lineages).^11^ Additional studies are required to confirm and replicate these observations. Studies have also delineated syndromic ASD genes where the diagnosis of autism co-occurs with other medical, neurological, cognitive, and psychiatric comorbidities,^42^ but studies comparing the cell-type enrichment of syndromic ASD genes versus non-syndromic ASD genes have, to our knowledge, not been conducted.

Gene-disrupting variants detected in association studies are either loss-of-function variants or dominant gain-of-function variants. While ASD-associated genes have been stratified into those preferentially disrupted by loss-of-function versus missense variants,^65^ this stratification has not been used to identify cell types with a preferential sensitivity for one class of variant over the other. Generally, due to a lack of statistical power, association studies have aggregated all classes of variants at the gene level to reach genome-wide significance.^11^^,^^65^

In conclusion, while analyses of ASD-associated gene-disrupting variants suggest cellular convergence at the level of broad cell classes, establishing links between different categories of ASD-associated genes and specific cell types remains challenging.

### Functional annotation of genes using transcriptomic architectures of cell types

Assuming cell type represents a functional unit of brain structure, we next seek to predict what cell types are disrupted when a particular gene is mutated. One might expect that genes with high AUC values (specificity/sensitivity as defined in section transcriptomic architecture of cell types) are most likely to selectively disrupt the associated cell type. While this idea is intuitive, it has been primarily explored in the context of conditions other than autism. For instance, this hypothesis is supported by numerous examples in genetic forms of blindness, where mutations in rod- or cone-specific genes lead to selective degeneration of these cells.^66^^,^^67^^,^^68^ However, there are notable counterexamples in neurological disorders, including genes that are ubiquitous yet whose mutations primarily affect a single cell type. As an example, *SMN1* is expressed in nearly all cells, but its deficiency in spinal muscular atrophy (SMA) primarily affects motor neurons. This selectivity is not fully understood, but it is likely linked to the specific functions of SMN in these cells, particularly in pre-mRNA splicing, and to the high energetic demands of motor neurons.^69^

**Figure 3:**
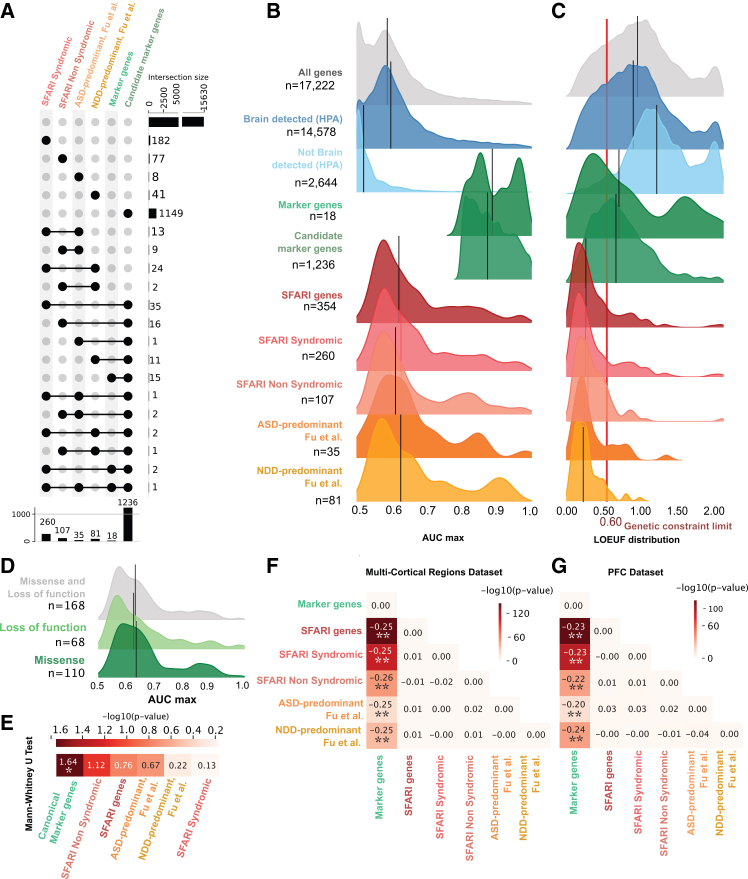
Marker genes and ASD-associated genes differ in cell-type specificity and genetic constraints (A–C) (A) Upset plot depicting the number of genes shared across different gene sets, including 18 canonical marker genes, 1,236 candidate marker genes, and genes linked to ASD: 260 SFARI syndromic, 107 SFARI non-syndromic,^42^ 35 ASD-predominant, and 81 NDD-predominant.^11^ Ridge plots showing the distribution of the AUC_max_ across all 19 cell types for (B) and LOEUF values for (C), computed for the whole protein-coding genome, genes detected in the brain (blue) or not (light blue) by bulk transcriptomic data (HPA^70^^,^^71^), and the gene sets described in (A). Black lines depict the median of each distribution. The red line represents the limit of genes under genetic constraint (LOEUF < 0.6). CT, cell type. (D) Ridge plots displaying the distribution of AUC_max_ values for ASD-associated genes primarily impacted by loss-of-function variants, missense variants, or both loss-of-function and missense variants.^65^ (E) –log_10_(*p* values) from two-sided Mann-Whitney U tests comparing the distributions of AUC_max_ between the Allen multi-region dataset and the adult PFC^72^ dataset computed separately for each gene set. There is no significant differences between the distributions of AUC_max_, in both datasets except for the marker-gene set. Overall, low AUC_max_ values of ASD-related genes are conserved in both datasets. (F and G) Heatmaps of −log_10_(*p* value) from two-sided Mann-Whitney U tests comparing AUC_max_ distributions across gene sets within the Allen multi-region dataset (F) and within the adult PFC dataset^72^ (G). The color scale represents −log10(*p* value), with more saturated colors indicating stronger statistical significance. Numbers within cells denote the difference in medians between the two compared gene sets. Asterisks mark significant comparisons: ∗*p* < 0.05, ∗∗*p* < 0.01. Within each dataset, the different stratifications of autism-associated genes (SFARI syndromic/non-syndromic, ASD predominant, NDD predominant) do not differ significantly from one another. In contrast, the marker-gene set (which includes both canonical and candidate marker genes) differs significantly from each ASD-associated category, and this pattern replicates in both datasets.

The relationship between cell-type specificity, pleiotropy, and genetic constraint across genes is still unclear. As described above, ASD-associated genes are broadly enriched in glutamatergic and GABAergic classes.^11^^,^^12^ However, at the subclass and subtype levels, functional enrichment studies fail to converge, likely because ASD-associated genes are pleiotropic and functional vulnerabilities cannot be readily predicted from expression profiles alone. To better characterize the cell-type specificity and sensitivity of ASD-associated genes, we computed their AUC_max_ and compared them to candidate marker genes. The candidate marker set includes approximately 1,200 genes that are highly specific for one cell subclass (candidate marker genes, median = 0.87; and canonical marker genes, median = 0.89). Although these 1,200 cortical candidate marker genes showed high levels of specificity to predict cortical cell types/clusters, they only represented a fraction of genes implicated in ASD (Figure 3A). Among the 405 ASD-associated^42^ genes described in the previous section, 354 genes were detected in the Allen Human Brain Multiple Cortical Areas dataset,^25^ and only 13.5% were candidate marker genes with AUC_max_ > 0.8 (sensitivity/specificity). Overall, while ASD-associated genes showed low cell-type specificity with a median AUC = 0.62 (Figure 3B), AUC was still higher than randomly sampled genes (*p* = 10^−6^). In a few cases, their AUC was close to 0.5, suggesting a complete lack of cell-type specificity. These results highlight the fact that ASD-associated genes tend to be expressed in multiple cell types, suggesting pleiotropic effects. Expression profiles alone may therefore not be sufficient to infer cellular vulnerabilities.

In addition, genetic constraint (measured by LOEUF) was significantly lower for candidate marker genes compared to ASD-associated genes (*p* = 10^−60^; Figure 3C). Approximately 54% of the candidate marker genes were not intolerant to haploinsufficiency (LOEUF > 0.6). One explanation could be that variants disrupting genes specific to one cell type are more easily compensated for by other biological processes. Another explanation might be that the candidate marker genes identified here are based on adult cortical cell types, whereas ASD-associated genes show stronger expression in the prenatal brain, which has distinct marker genes and developmental trajectories. Genes specific to transient or developing cell populations may therefore show higher constraint and stronger overlap with ASD-associated genes.

Together, these findings suggest that genetic constraint and cell-type specificity may vary across subsets of ASD-associated genes. We therefore investigated whether four previously published categories of genes exhibit distinct patterns of cellular specificity: SFARI syndromic, SFARI non-syndromic,^42^ ASD predominant, and NDD predominant.^11^ These four categories showed similar low AUC_max_ distributions, suggesting consistent low levels of cell-type specificity (Figure 3B).

Beyond clinical categories, the type of genetic variant could also influence patterns of cellular convergence. We therefore analyzed whether genes preferentially impacted by missense or loss-of-function variants displayed distinct cell-type specificity. We used the stratification from Coe et al.^65^ The “Missense” gene set included genes classified as “missense” and “MIS30,” while the “Loss-of-function” gene set included those classified as “likely gene disruptive (LGD)” and “LGD and missense” (Figure 3D). Both groups showed similar low specificity, with median AUC_max_ values of 0.63 and 0.62, respectively.

To assess the robustness of these observations, we replicated the analysis in an independent snRNA-seq dataset from the human prefrontal cortex (PFC),^72^ restricting the analysis to adult individuals. Patterns of AUC_max_ across gene groups were highly consistent between datasets (Figures 3E–3G).

In summary, genes associated with autism and related NDDs are not enriched for marker genes and tend to show broad expression. This is consistent with previous reports showing that genes under genetic constraint with large effects on neurodevelopment^49^ have lower tissue specificity.^17^^,^^50^

### Convergence between genetic liability of autism and transcriptomic studies in *postmortem* brains

It has been challenging to reconcile transcriptomic variations observed in *postmortem* ASD brains with findings from genetic association studies.^30^^,^^73^ The largest transcriptomic studies have been performed in idiopathic ASD and converge on several key findings: (1) widespread transcriptomic changes across the cortex in ASD, exhibiting an anterior-to-posterior gradient, with the greatest differences in the primary visual cortex^74^; (2) substantial changes in gene expression across all cell types, most of which were cell-type specific, with a strong enrichment in upper-layer cell types^30^^,^^73^; and (3) upregulation of microglial, astrocyte, and neural-immune genes as well as profound downregulation of synaptic genes at the bulk and single-cell level.^30^^,^^73^^,^^74^

These findings raise many questions. Could the strong convergence of ASD *postmortem* brain studies on the upregulation of microglial, astrocyte, and neural-immune genes be due to the secondary effects of lifelong ASD symptoms in samples primarily collected from adults? Such convergence is intriguing given the heterogeneity of idiopathic ASD and the fact that transcriptomic studies in *postmortem* brains of 15q11-q13 duplications (a variant strongly associated with ASD) also converged on alteration of the same neural-immune transcriptional variations.^30^^,^^74^ These transcriptional variations observed in idiopathic autistic brains differ from the results of functional enrichment analyses performed for high-confidence autism genes. Notably, large-effect-size ASD-associated genes are not enriched in glial cells,^11^^,^^12^^,^^34^^,^^56^^,^^58^ and it remains unclear whether they affect specific subclasses of neurons (such as activated SST interneurons^30^) since enrichment analyses have not been performed at such levels of granularity. Whether the preferential involvement of upper-layer neurons observed in transcriptional studies converges or diverges with functional enrichment analyses of ASD-associated genes also remains unclear.

A few studies tried to reconcile transcriptomic variations observed in *postmortem* ASD brains with findings from genetic association studies.^30^^,^^73^ At the bulk tissue level, only a small overlap (5%) was observed between 253 ASD SFARI genes and 487 DE genes in autistic brains.^73^ This may partly be explained by the small sample size of this study (15 cases and 16 controls), which only provided power to identify differential expression for genes with substantial changes in expression (well above 1–2 standard deviations). It is also possible that only a few of these SFARI genes were altered in the 15 cases. It is therefore likely that differential expression will be identified in a much larger proportion of genes with larger sample sizes. Given that transcriptomic changes are cell-type specific, alternative approaches have been investigated to elucidate the relationship between genes identified through genetic association studies and transcriptional signatures observed in specific cell types in autistic brains. Researchers have identified 217 “regulons,” defined as a module of cell-type-specific ASD-DE genes overlapping with known transcriptional factor networks.^30^ This approach showed that many high-confidence ASD-associated genes were candidate drivers of 15% of these observed cell-type-specific regulons. While most of these ASD-gene regulons are DE in neuronal cell types (both excitatory and inhibitory classes), a few of them (seven) are astrocyte and oligodendrocyte precursor specific. Although these overlaps are still limited, they indicate that the genetic risk for the condition may, to some extent, align with the transcriptomic changes seen in glial cells. However, validating robustly the impact of ASD-associated variants on brain cell types would require brain tissue from individuals carrying variants in those high-confidence ASD-associated genes. Except for the study of the 15q13 duplication, very few brains harboring high-risk variants are available in brain banks overall, and their effects on the transcriptome and distribution of cell types have not yet been investigated.

These divergences highlight the importance of extending our knowledge beyond severe high-impact ASD-/NDD-associated genes identified through an excess of *de novo* variants. Variants with more moderate effects may prove more consistent with transcriptomic studies.

Divergence could also point toward other levels of biological convergence (beyond cellular). The concept of cellular convergence in genetic risk is worth reconsidering, as the biology of autism may be influenced by mechanisms operating at broader molecular or “meta-cellular” levels such as large-scale brain networks.

## Promises and challenges of Perturb-seq and foundation models for predicting the effects of ASD-associated variants

### Challenges of using Perturb-seq to mimic ASD-associated variants

**Figure 4:**
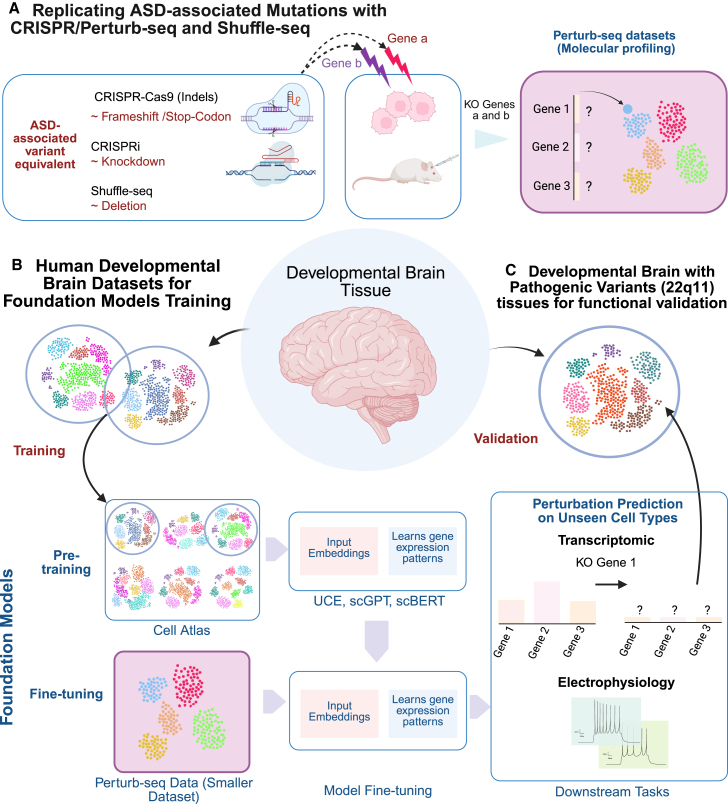
Framework for leveraging Perturb-seq experiments and foundation models in predicting the effects of ASD-associated variants (A) CRISPR-induced genetic disruptions, mimicking ASD-associated variants, are performed both *in vitro* and *in vivo*. Molecular profiling of these genetically perturbed cells generates Perturb-seq datasets, providing transcriptomic profiles of cellular responses to these genetic perturbations. CRISPR-Cas9 functionally approximates an indel, CRISPRi creates a knockdown, and Shuffle-seq functionally approximates a deletion, although these equivalences have limitations. (B) Human developmental brain tissues should be prioritized for the pre-training of foundation models, utilizing large, broad, and unlabeled datasets. During pre-training, the model learns complex co-expression patterns among genes across different cell types and species. Once pre-trained, the model can be fine-tuned on smaller datasets, such as Perturb-seq data (generated in A), to specialize in specific downstream tasks. This fine-tuned model can predict the effects of perturbations on both transcriptomic profiles and electrophysiological properties of cells. (C) Results from foundation models should be validated using *postmortem* developmental tissues from human samples carrying specific pathogenic variants, ensuring the accuracy of the predictions. The illustration of foundation models’ structure is inspired by Cui et al.^81^

Interpreting the effects of ASD-associated variants on brain cell-type transcriptomes, electrophysiology, and morphology is challenging, in part due to the inability to experimentally validate, at scale, their effects on human brain structure and function. The development of multiplexed gene-expression perturbation methods based on clustered regularly interspaced short palindromic repeats (CRISPR), including Perturb-seq and CRISPR droplet sequencing (CROP-seq), has significantly advanced the field.^18^^,^^20^^,^^75^ These pooled screening platforms couple CRISPR-based perturbations with scRNA-seq, enabling scalable interrogation of haploinsufficiency across multiple genes. In particular, Perturb-seq is compatible with a variety of CRISPR effectors (e.g., CRISPRi/a, base editing) and has been more widely used for ASD-associated genes. Perturb-seq can be applied to study cells *in vitro*^76^ as well as *in vivo*^77^ (Figure 4A). The integration of Perturb-seq with advances in single-cell genomics technologies has enabled massively parallel analyses of cellular and molecular consequences of many loss-of-function mutations in neural progenitor cells, neurons, and glia.^77^^,^^78^^,^^79^^,^^80^

Most *in vivo* Perturb-seq experiments have been conducted in mouse models. While these results are often considered translatable to human neuronal classes, given the broad conservation of brain cell types across species, significant transcriptomic differences exist. For example, the Allen Institute’s Primary Motor Cortex taxonomy identified 125 DE genes for human LAMP5 interneurons, yet only 51 of these are also DE in mice. Similarly, among 87 DE genes characterizing human VIP interneurons, only 31 show differential expression in the mouse counterparts. These findings underscore the importance of caution when extrapolating transcriptomic results from mouse models to human brain cell types and disorder mechanisms.

Of relevance to autism and related NDDs, a study perturbing 35 autism-associated genes in mouse *in vivo* identified molecular and cellular phenotypes across many cell types of the mammalian brain.^77^ Notably, the study highlights two key patterns: some genes exert highly cell-type-specific effects when mutated, while others—despite being ubiquitously expressed—also produce distinct, cell-type-specific consequences. For instance, consistent with the reported differential expression of *Gatad2b* in oligodendrocytes,^70^^,^^71^ perturbation of this gene led to an oligodendrocyte-specific phenotype.^77^ This finding aligns with the assumption that the cell type mostly impacted by a gene-disrupting variant is the one where the gene is specifically/preferentially expressed. Counterexamples have also been reported, such as *Ank2*, which encodes ankyrin-B, a protein that organizes the cell’s cytoskeleton and cellular membrane. Despite the broad expression of *Ank2* in excitatory and inhibitory neurons as well as in glial cells, its perturbation specifically impacts GABAergic interneurons’ transcriptome.^77^ This result could suggest a lack of compensatory mechanisms in interneurons or an interaction of *Ank2* mutated transcript with interneurons’ transcription factors. Thus, Perturb-seq studies highlight the challenge of using gene-expression maps from healthy brains to predict cell-type alterations due to gene-disrupting variants, even at the basic level of transcriptomic consequences. Jin et al. further examined the overlap between Perturb-seq-induced DE genes and DE genes from *postmortem* ASD brains.^77^ They identified two genes, *SST* in interneurons and *NRN1* in excitatory neurons, both of which showed decreased expression in ASD patients and were likewise significantly decreased in expression across Perturb-seq experiments, albeit with different effect sizes. However, current evidence remains insufficient to conclude that Perturb-seq recapitulates *postmortem* transcriptional signatures more faithfully than expression-enrichment approaches.

Perturb-seq- and CROP-seq-based disruptions of autism-associated genes have also been performed in human neural cell lines^78^^,^^79^ and brain organoids.^55^^,^^80^ Human models using induced pluripotent stem cells (iPSCs) and organoids enable researchers to track the fate of perturbed cells over time and examine how gene disruptions alter their differentiation trajectories. These studies consistently found that subsets of ASD-associated genes impair excitatory and inhibitory neurogenesis by delaying or accelerating cell differentiation. In one large-scale organoid experiment targeting 36 high-risk ASD-associated genes, the investigators examined cell-type-specific effects of these perturbations.^55^ They highlighted that L2/3 excitatory neurons were the most impacted subclass and were even depleted in most perturbations.

However, most of these studies filtered for ASD-associated genes with a predicted role in transcriptomic regulation. In a more recent study using human iPSCs to generate a model of excitatory corticogenesis,^82^ the authors repressed the expression of 87 ASD-associated genes, selected solely based on their statistical association in Satterstrom et al.,^12^ without prior assumptions about their biological function. Their findings further support the observation that disrupted excitatory neurogenesis is a key point of convergence among ASD-associated genes. Additionally, the study helped uncover specific molecular mechanisms, including impaired microtubule-related function. However, further work is needed to understand how these *in vitro* findings relate to neurodevelopmental phenotypes in human patients.

While Perturb-seq and CROP-seq are powerful tools for modeling gene function, their results must be interpreted carefully when drawing parallels to pathogenic variants involved in ASD. While CRISPR technologies generate loss-of-function mutations through either Cas9-induced indels or CRISPR interference knockdown (CRISPRi), these experimental disruptions do not precisely mimic naturally occurring pathogenic variants found in ASD patients. Cas9-induced mutations typically introduce frameshifts or premature stop codons at random positions within the gene, leading to potential production of truncated transcripts or altered proteins with unpredictable effects. In contrast, the position of disorder-associated variants within a gene contributes critically to their functional consequences. As such, the random distribution of Cas9-mediated indels may result in incomplete loss of function or unanticipated downstream effects not representative of those seen in patients. CRISPRi, which downregulates gene expression by blocking transcription, may offer a more controlled and uniform reduction in expression, akin to a gene deletion. However, even this approach may fail to fully capture the complexity of dosage-sensitive mutations. Indeed, CRISPRi typically results in partial gene inactivation, as the genomic sequence remains intact and may still be accessible to transcription factors and regulatory elements. Since most ASD-associated mutations are heterozygous loss of function, the target with CRISPRi would be a ∼50% reduction in gene expression, but achieving this precise degree of knockdown is experimentally challenging. Moreover, because CRISPRi does not physically remove the DNA, the manipulation alone does not replicate the potential *cis*-regulatory or topological disruptions that an actual genomic deletion might cause, particularly those affecting nearby genes or long-range chromatin interactions. To address this issue, new methods such as Genome-Shuffle-seq can introduce large structural variants.^21^ Overall, when used to model ASD-associated variants, CRISPR-/CROP-/Shuffle-based perturbations should be interpreted as approximations rather than direct analogs of the mutations observed in affected individuals.

Additionally, understanding the effects of genetic variants on brain development requires precise control over both timing and cell-type specificity, which hinges on advancements in CRISPR technology. Current techniques lack temporal specificity, as methods are largely dependent on constitutive expression of CRISPR effectors. Another challenge is cell diversity. The most recent brain atlases have been successful at identifying hundreds of cell types^4^^,^^25^^,^^83^ within unique regions (primary motor cortex, M1). Achieving sufficient statistical power to predict the effects of multiple variants across various cell types is challenging, as it depends on the number of cells analyzed and the magnitude of the effects. Detecting a single-gene differential expression with a 2-fold change across 14 cell types requires sequencing 2,100 cells,^18^^,^^19^ implying that mapping the entire genome would require 44.1 million cells—or even more for finer cell-type granularity (5,000 subclasses). This highlights the scale of the challenge in predicting genome-wide perturbations at the subclass level. To partially address these limitations, other methods, such as Targeted Perturb-seq (TAP-seq), increase the sensitivity of single-cell measurements and enable the detection of modest expression changes in a predefined subset of genes of interest.^75^

Furthermore, Perturb-seq captures acute transcriptional responses to genetic perturbations. This includes the induction of immediate-early genes (IEGs), which reflect transient activation states rather than stable regulatory programs. In contrast, the transcriptional effects of ASD-associated variants are likely to arise from long-term developmental or homeostatic disruptions.

Rare (CNVs) and common variants associated with ASD often affect combinations of genes.^84^ However, the number of individual genes and gene combinations tested so far in Perturb-seq experiments remains a small fraction of the vast array of possible interactions. So far, datasets containing around 1,100/1,500 perturbations of the genome have been generated in retinal pigment epithelial cells and myeloid leukemia,^19^ with a single genomic background. However, real ASD patients exhibit a wide diversity of genomic backgrounds that influence disorder etiology through compensatory or enhancing mechanisms. This complexity is not yet captured by Perturb-seq experiments, which are typically conducted within a single genomic background.

Due to the limitations described above, deep-learning/foundation models trained on vast single-nucleus datasets could help predict the effects of gene-disrupting variants^22^^,^^85^ across cell types.

### Promises and limitations of foundation models

Deep-learning models can help forecast how genetic variants affect cell types. Among these models, single-cell foundation models are pre-trained on extensive single-cell atlases to learn general representations of cells based on their gene-expression profiles. Once pre-trained, these models can be applied directly to perform different tasks on datasets irrespective of their size, without the need for retraining. (This ability to be used immediately, without retraining, is referred to as zero-shot learning.) This makes foundation models broadly accessible, enabling a wider range of laboratories, including those with limited data, to use them for perturbation prediction.

Major foundation models for single-cell analysis include scGPT, scBERT, and Universal Cell Embedding (UCE),^81^^,^^86^^,^^87^ which are pre-trained on single-cell/single-nucleus data. They differ in their design and their approach to handle perturbation tasks. UCE emphasizes zero-shot usability, enabling embeddings of new datasets—Perturb-seq datasets or data from different species—without retraining. In contrast, scBERT is designed to learn gene-gene relationships from masked inputs, and scGPT is a generative model capable of predicting expression changes after perturbations. Both scBERT and scGPT require fine-tuning on the new dataset for perturbation-specific tasks and are not zero shot. Importantly, recent benchmarking indicates that deep-learning-based approaches (including the three types of models) for predicting gene perturbation effects do not yet outperform simple linear models.^88^

Models described above are trained on transcriptomic datasets without genetic information and, as a result, are not capable of predicting the effects of gene-disrupting variants and their combinations (e.g., a given variant on different polygenic backgrounds). A second category of foundation models is pre-trained directly on Perturb-seq data and is designed to capture the effects of combinatorial gene perturbations. For example, graph-enhanced gene activation and repression simulator (GEARS)^85^ has demonstrated relatively good performance in predicting transcriptome-wide effects of gene-pair-disrupting mutations (the Pearson correlation between the gene expression predicted by the model and observed experimentally was r = 0.55). Validated through combinatorial cell fitness screens, GEARS enhances the prediction of synergies and suppression effects between gene mutations.

Foundation models could be leveraged to predict the impact of variants on other cellular phenotypes, such as electrophysiology^89^ and connectivity patterns. A foundation model has been developed and trained to predict neuronal activity in response to visual stimuli.^89^ It has unexpectedly been demonstrated to be also able to predict morphological and connectivity patterns. Specifically, this model can uncover complex relationships between neuronal function and connectivity. Such models (and the relationship between connectivity and electrophysiology that they learn) could potentially be leveraged to analyze Perturb-seq data, thereby providing deeper insights into the broader impact of genetic variants on diverse cellular phenotypes. Neuron-editing frameworks, which aim to predict the impact of mutations or variants in cell types that were not included during model training, stand to benefit greatly from these foundation models and the resulting large-scale databases. This could reveal whether modifying a marker gene in a specific cell type has widespread effects or whether mutations in pleiotropic genes broadly impact the transcriptome. Beyond transcription, these advances will enhance our understanding of genetic variants’ influence on the epigenome and electrophysiology. Ultimately, integrating Perturb-seq data with deep learning and universal cell/gene representations could provide a powerful approach to assessing genetic variants’ functional impact on cell phenotypes.

Using foundation models to better understand the etiology of ASD presents unique challenges, since these models need to be trained on human developing brain tissues at various stages of development. Autism has been modeled using other species, but it is unclear how the effects observed on mice could be translated to the human brain. An important question to address is what datasets are required to effectively train/fine-tune these models (Figure 4B) and validate their predictions (Figure 4C). Introducing developmental datasets with well-known variants (22q11) into those frameworks could offer a powerful insight into the biology of ASD. Although existing foundation models typically represent individual cells,^86^ the interplay between different cells plays a fundamental role in the developing brain, and understanding ASD requires exploring how cellular ecosystems function within the constraints of the human brain architecture beyond the individual cell level.

## Conclusions

To date, the functional interpretation of ASD-associated gene-disrupting variants has primarily been addressed through enrichment methods, which require the assignment of genes to specific cell types based largely on their expression specificity. However, the systematic characterization of sensitivity and specificity across ASD-associated genes demonstrates that many of them exhibit modest cell-type specificity. Therefore, despite progress in mapping the genetic architectures of autism and related NDDs, neither the biochemical pathways nor cell-type-specific expression appears to point toward a clear point of convergence. Co-expression enrichments of causal genes implicate pathways and cell types that differ from those observed in *postmortem* brain studies, underscoring the gap between genetic association and disease-state biology. There is a need to move beyond gene-level associations to get true mechanistic insights into the consequences of patient-specific variants, since different variants in the same ASD-associated gene can have variable molecular effects, distinct cell-type liabilities, and divergent impacts on development. Collapsing them into a single experimental model obscures critical biology. Due to the scale of the challenge, foundation models trained on single-cell data and perturbations will be essential to extrapolate findings to untested variants, contexts, and combinations.

## Acknowledgments

This project was supported by the 10.13039/100000025National Institute of Mental Health and 10.13039/100000065National Institute of Neurological Disorders and Stroke with grant numbers U01MH130962, R01NS123263, and R01MH128364 (to T.N.) as well as gifts from the Esther A. & Joseph Klingenstein Fund, the 10.13039/100010319Shurl and Kay Curci Foundation, the 10.13039/100006064Sontag Foundation, and the 10.13039/100010246William K. Bowes, Jr. Foundation. T.N. is a New York Stem Cell Foundation Robertson Neuroscience Investigator. This research was enabled by support provided by Calcul Quebec (http://www.calculquebec.ca) and the 10.13039/501100021202Digital Research Alliance of Canada (https://www.alliancecan.ca/). S.J. is a recipient of a chair from the 10.13039/100013367Jeanne and Jean-Louis Lévesque Foundation. This work was also supported by grants from the Canadian Institutes of Health Research and the Fondation Courtois.

We thank Dr. Stephan J. Sanders for critical reading of this manuscript. The creation of figures involved the use of BioRender software.

## Author contributions

Conceptualization, A.D., S.J., and T.N.; literature curation, A.D.; figure creation, A.D., G.H., and T.R.; data analysis, A.D. and T.R.; data interpretation, A.D., S.J., T.R., and T.N.; writing – manuscript draft, A.D., G.H., S.J., T.R., and T.N.; writing – review & editing, A.D., S.J., and T.N.; supervision, S.J. and T.N.; funding acquisition, S.J. and T.N.

## Declaration of interests

The authors declare no competing interests.
